# Cryoablation for Ventricular Tachycardia Originating from Anterior Papillary Muscle of Left Ventricle Guided by Intracardiac Echocardiography

**DOI:** 10.1155/2017/9734795

**Published:** 2017-04-20

**Authors:** Ibrahim Marai, Nizar Andria, Osnat Gurevitz

**Affiliations:** ^1^Division of Pacing and Electrophysiology, Cardiovascular Center, Padeh-Poriya Hospital, Tiberias, Israel; ^2^Davidai Arrhythmia Center, Heart Center, Sheba Medical Center, Tel Hashomer, Israel

## Abstract

Papillary muscles (PMs) were reported to be origin of ventricular arrhythmia (VA). Radiofrequency (RF) ablation was reported to be acutely effective in eliminating VA. However, the recurrence rate is high. Recently, cryoablation guided by intracardiac echocardiography, 3-dimensional mapping system, and image integration was introduced as alternative strategy for this challenging ablation. We present a case of ventricular tachycardia originating from anterior PM of left ventricle treated by cryoablation guided only by intracardiac echocardiography.

## 1. Introduction

Papillary muscles (PMs) were reported to be origin of ventricular arrhythmia (VA). Radiofrequency (RF) ablation was reported to be acutely effective in eliminating VA [1]. However, the recurrence rate is high. Recently, cryoablation was introduced as alternative strategy for this challenging ablation.

## 2. Case Report

A 75-year-old woman was admitted because of wide complex tachycardia with right bundle branch block pattern and right inferior axis. She was treated with adenosine and verapamil with no effect. Sinus rhythm was achieved by DC shock. Transthoracic echocardiography revealed normal systolic function without wall motion abnormalities, moderate concentric hypertrophy, and severe aortic stenosis. Coronary angiography showed nonsignificant stenosis of left anterior descending artery.

**Figure 1:**
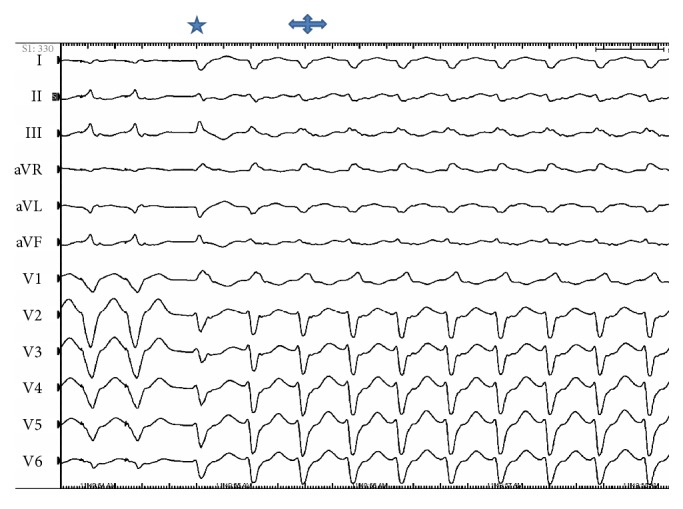
ECG showing spontaneous subtle change in QRS morphology after induction of ventricular tachycardia. The first beat (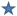) of tachycardia resembles the clinical VT. The second beat (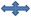) of tachycardia and the rest of beats have the same pattern but with subtle difference indicating most probably different exit point from the papillary muscle.

**Figure 2:**
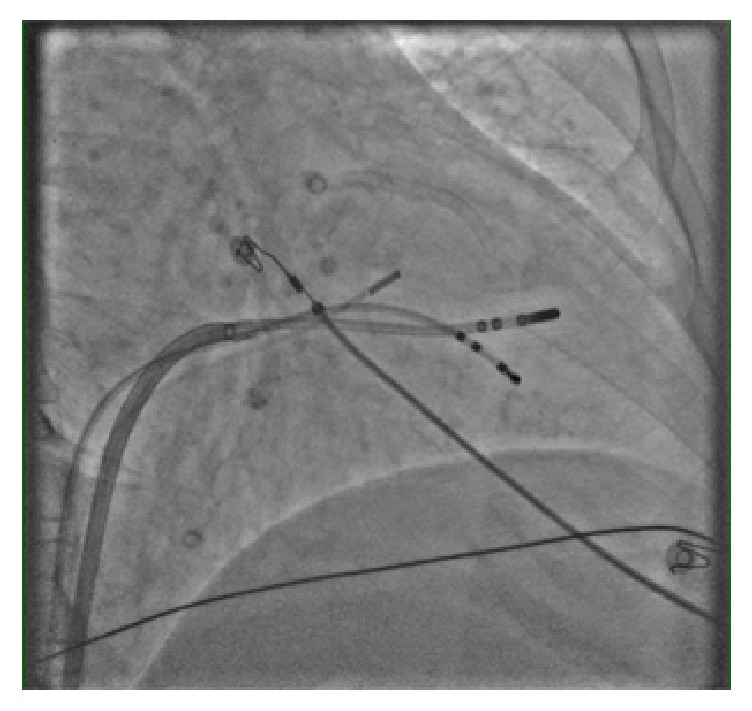
Fluoroscopy showing the position of cryocatheter at anterior papillary muscle in RAO projection.

**Figure 3:**
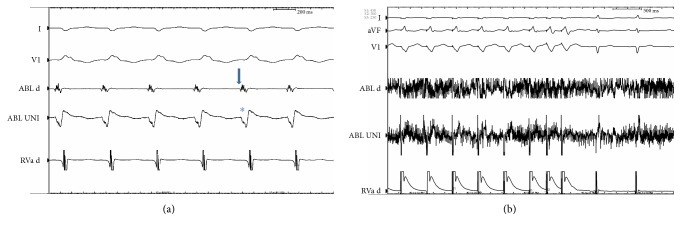
(a) Intracardiac electrogram showing bipolar prepotential preceding the surface QRS by 35 milliseconds (arrow) and unipolar QS pattern (asterisk) recorded at the ablation site during ventricular tachycardia. (b) Programmed stimulation during cryoenergy application did not induce ventricular arrhythmia. The noise in the ablation channel (ABL) is due to ice artifact.

**Figure 4:**
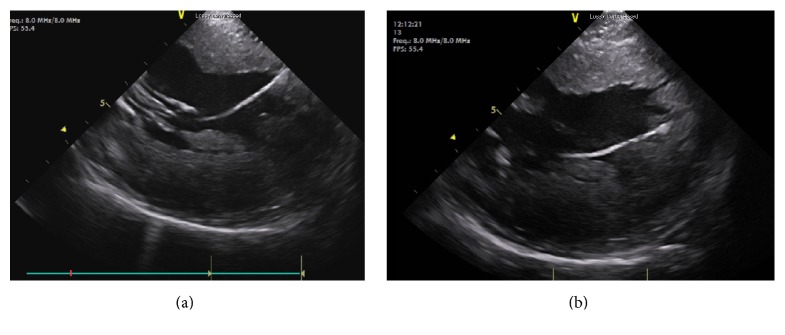
(a) Intracardiac echocardiography showing the tip of the catheter at the anterior side of anterior papillary muscle. (b) Intracardiac echocardiography showing the tip of the catheter at the posterior side of anterior papillary muscle. Ice formation is shown at the tip of cryocatheter during cryoenergy application.

The patient underwent electrophysiological study. The procedure was performed under conscious sedation. Standard multielectrode catheters were placed in the His bundle region, coronary sinus, and RV apex through the right and left femoral veins. Ventricular tachycardia (VT) resembling the clinical tachycardia was easily induced by programmed electrical stimulation from the RV apex. The arrhythmia was nonsustained and became sustained after isoproterenol infusion. Spontaneous changes in QRS morphology were observed (Figure 1). 6 mm cryoablation (Freezor MAX 3, Medtronic, Inc., Minneapolis, MN) catheter was advanced into the left ventricle (LV) via deflectable sheath through a transseptal and transmitral approach. Intravenous heparin was administered to maintain an activated clotting time of 300 s. A 2-dimensional intracardiac echo (ICE) probe was advanced via femoral vein and localized at right ventricular outflow tract to visualize real-time location of cryocatheter during mapping and ablation. Activation mapping by the cryocatheter revealed that the site of origin was at anterior PM of LV as confirmed by ICE (Figure 2). No Purkinje potentials were recorded at the ablation site during sinus rhythm. Prepotential preceding the surface QRS by 35 milliseconds was recorded at the ablation site during VT (Figure 3(a)). The VT was mechanically interrupted by the contact of the cryocatheter with the base of anterior PM but nonsustained VT recurred until freezing was started. During and after the first application, VA could not be induced with and without isoproterenol (Figure 3(b)). Another 2 applications were done at both sides of anterior PM (Figures 4(a) and 4(b)). Finally, 3 applications up to 240 s with freeze-thaw-freeze cycles were done. During 45 minutes after the end applications, no VA could be induced. No mitral regurgitation was seen at the end of the procedure. Three-dimensional mapping system was not used. The patient was free of VA and free of mitral regurgitation during follow-up of 4 months.

## 3. Discussion

Catheter ablation of PM VAs is challenging compared with the other VA in the LV [1]. The complex anatomic structure of PMs may lead to multiple QRS morphologies and causes difficulties in catheter manipulation and stabilization during mapping and ablation [2]. The maneuverability of the catheter is more challenging if a deflectable sheath is not employed. This can explain the high recurrence rate after RF ablation.

Yamada et al. [2] reported their experience of RF ablation for VA originating from LV PM using 3-dimensional (3D) electroanatomic mapping system. In all (*n* = 19) patients, transthoracic and intracardiac echocardiography and left ventriculography revealed that the successful ablation sites were localized at the base of the PMs in the LV. Additional ablation targeting a relatively wide area around the first RF lesion was required to completely eliminate the VAs in all patients. Multiple QRS morphologies were seen spontaneously and after the ablation in 47% of patients. RF lesions on both sides of the PMs were required in these patients. During the follow-up, VAs recurred in 58% of patients.

Rivera et al. [3] performed focal cryoablation, up to 240 s with freeze-thaw-freeze using an 8 mm cryoablation catheter via a transmitral approach in 10 patents with PM VAs. In this study, VAs foci were localized to anterior or posterior PM using 3D mapping system, multidetector computed tomography (CT), and ICE. Termination of VA was observed in all patients during ablation. VA recurred in 1 (10%) patient.

In another study, Rivera et al. [4] compared outcomes and complications of catheter ablation of VA from the PM of the LV (localized using 3D mapping system, multidetector CT, and ICE) with either cryoenergy or RF. Acute success rate was 100% for cryoenergy and 78% for RF radiofrequency (*P* = 0.08). Catheter stability was achieved in all patients treated with cryoenergy and only in 25% patients treated with RF (*P* = 0.001). Incidence of multiple VA morphologies was observed in 77.7% patients treated with RF, whereas none was observed in those treated with cryoenergy (*P* = 0.001). VA recurrence at 6 months' follow-up was 0% for cryoablation and 44% for RF (*P* = 0.03).

The acute and long-term advantage of cryocatheter over RF catheter is stability during energy application. Keeping good contact of RF catheter with tissue during the whole period of RF application is very difficult because of contractility of both LV and PM. In addition, ablation of wide area on both sides of the PMs might be required to completely eliminate VA as changes in QRS morphology and a shift in the breakout site to the opposite side of the PM can occur spontaneously and after the ablation [2]. During freezing, within few seconds the cryocatheter adheres to tissue leading to maximal stability during all the time of application as showed nicely by Rivera et al. [4]. In our case, during the first application we tried to induce VA by pacing from RV apex without affecting stability of cryocatheter.

The uniqueness of our case is that cryoablation was guided by ICE without 3D mapping system or CT image integration. Cryocatheter can be integrated with the NavX system but not with the CARTO system. So we could not use 3D mapping system as we have only the CARTO system in our laboratory. Many centers have only 1 3D mapping system. In addition, ICE can help to localize the origin of arrhythmia, although differentiating ventricular arrhythmias originating from the papillary muscle and the fascicles is challenging and not always possible [5]. It can facilitate 3D electroanatomical mapping allowing for real-time creation of precise geometries of cardiac chambers and endocavitary structures [6]. Nakahara et al. reported that multipolar catheters may be helpful for identifying tachycardia origins arising from the posterior papillary muscle [7]. Thus, the feasibility of performing cryoablation of papillary muscle with the guidance of ICE without 3D mapping system is very important.

In summary, cryoablation guided only by ICE of VA originating from papillary muscle is feasible and should be considered as alternative strategy in this challenging condition.
